# Stigmatization and Healthcare Inequity Perceived by Individuals Served by Homeless Shelters in an Appalachian City

**DOI:** 10.13023/jah.0704.04

**Published:** 2025-12-01

**Authors:** Molly Powney, James M. Mears, Reagan Sharp, Lisa Calderwood

**Affiliations:** West Virginia University; Charleston Area Medical Center; West Virginia University; CAMC Insitute for Academic Medicine

**Keywords:** Appalachia, bias, healthcare barriers, homeless, minority groups, perceived stigma, unhoused

## Abstract

**Introduction:**

People experiencing homelessness are medically vulnerable and suffer disproportionately from chronic physical and mental health conditions. Evidence-based standards to inform decisions surrounding care would be valuable for this population. Studies exploring how homeless individuals in Appalachia perceive their interactions with the healthcare system remain limited, however.

**Purpose:**

The purpose of this study is to assess perceptions of the healthcare system among homeless individuals served by temporary housing shelters in an Appalachian city.

**Methods:**

A voluntary anonymous survey was administered to residents at three shelters in Charleston, West Virginia (WV) from September 2023 – March 2024. Responses were collected utilizing REDCap by a medical student interest group that regularly provided services at the shelters.

**Results:**

Of 108 participants, 23.2% “agreed” or “strongly agreed” that they feel judged by healthcare workers, and 23.2% agreed/strongly agreed that they feel healthcare workers treat them differently from the non-homeless people in the hospital. Similarly, 19.4% agreed/strongly agreed that they do not like going to the hospital because of the way they are treated. Respondents with a history of a mental health diagnosis reported significantly higher levels of stigma or poor experience with providers (*p* =0.026). There was no significant difference in perceived stigma/judgement based on race, incarceration history, or employment status.

**Implications:**

Perceived inequity in health care was prevalent amongst the homeless individuals surveyed. Survey participants with a history of mental health diagnoses were more likely to report dissatisfaction with care. More studies are needed to determine the impact of perceived stigmatization on healthcare disparities in the homeless population in WV.

## INTRODUCTION

In the most recent Point-in-Time count (an annual nationwide effort conducted by the United States Department of Housing and Urban Development to estimate the number of unsheltered and sheltered people experiencing homelessness on a given night), approximately 771,480 people were experiencing homelessness on a single night in the United States (U.S.) in January 2024—the highest number recorded since national counts began in 2007. 1 This figure indicated an 18% increase from the previous year. Of those counted, about 36% were unsheltered, while the remaining 64% were staying in emergency shelters or transitional housing. 1

Family and child homelessness increased sharply in the most recent surveillance data, with a 33% rise in children experiencing homelessness and a 39% increase in homelessness among families with children as compared with 2023.^1^ While veteran homelessness decreased nationally, racial and ethnic disparities persisted across the general homeless population. Black individuals comprise nearly 25% of the U.S. homeless population despite being only 13% of the general population, and Hispanic or Latino individuals make up roughly 30% of the homeless population but only 19–20% of the general population.^1^, ^2^ Over half of the nation's total homeless population reside in California, Florida, Illinois, Massachusetts, New York, Texas, or Washington.^2^

The causes of homelessness are multifactorial, involving a complex interplay of structural and individual-level contributors including lack of affordable housing, poverty, mental illness, substance use, criminal justice involvement, and insufficient government support.^3^, ^4^ Even in cities such as San Francisco, where governments allocated significant resources to address homelessness, outcomes have been modest due in part to inefficient program coordination and an overemphasis on temporary rather than preventive or long-term solutions.^5^, ^6^

Housing instability is closely tied to health. Lack of reliable shelter contributes to increased psychological stress, exposure to environmental hazards and violence, and disrupts care management and adherence to treatment plans. 7–9 Homeless individuals suffer disproportionately from chronic physical and mental health conditions and have a life expectancy 12 years shorter than the general U.S. population.^10^ Homeless individuals are more likely to suffer from mental illness, substance use disorders, obstructive lung disease, liver conditions, and kidney disease as compared with housed individuals.^11^ Experiencing homelessness can contribute to suboptimal and inefficient health care utilization and undertreated medical conditions that contribute to early morbidity and mortality.^11^ Access to primary care is limited for people experiencing homelessness, leading many to rely on emergency departments (EDs) for basic health needs. National studies have revealed that homeless individuals are three times more likely than the general population to visit an ED at least once within a year. 11, 12

Given common criticisms of reliance on EDs for non-emergent care, questions of fiduciary responsibility for uncompensated care, and poor access to services, evidence-based standards to inform decisions surrounding care would be valuable for this particularly vulnerable population. However, research exploring how homeless individuals perceive their health and interactions with the healthcare system remain limited in the U.S., potentially reflecting our national engagement with homeless individuals as a society and the challenges of working with homeless people as a research population.

Some qualitative studies have been conducted in Canada and Europe, where recurring themes include stigma, shame, healthcare system inflexibility, and inequity. 13 One U.S. study from Wisconsin identified that basic survival needs (e.g., food, shelter, water), transportation barriers, and poor provider empathy impede healthcare access among the homeless population.^14^ Another study focusing on homeless men highlighted experiences of discrimination, a desire for patient-centered care, and frustration with fragmented services.^15^ These findings are likely relevant to underserved regions of the U.S., including Appalachia, where research on homelessness and health care has been sparse and where unique societal and structural barriers shape healthcare experiences.

The National Alliance to End Homelessness estimated that 1,779 individuals were experiencing homelessness in West Virginia (WV) on any given night in 2024, including approximately 335 in Kanawha County, where the state capital of Charleston is located, and its surrounding counties (Putnam, Boone, and Clay).^16^,^17^ This particular region of WV has one of the highest population densities of unhoused and homeless individuals in the state.^16^,^17^ WV consistently ranks among the worst-performing states nationally in many health metrics and faces challenges to providing health care that are minimized in states with larger urban centers.^18^,^19^ Barriers including primary care and specialist provider shortages, geographic isolation, socioeconomic hardships, and mistrust of the healthcare system compound to delay patient care, fragment treatment, and often result in poorer health outcomes, such as higher rates of chronic disease, preventable illness, and early morbidity and mortality.^18^,^20^ This is especially important in populations facing intersecting barriers to health, such as the homeless population.

In response to the large homeless population in Charleston and the state of healthcare availability and health outcomes for the region as a whole, medical and community organizations have worked to fill service gaps. One such initiative is Charleston Homeless Street Medicine (CHASM), a nonprofit organization founded by students and faculty at the West Virginia University School of Medicine – Charleston Division. Modelled after Project Safety Net in Pittsburgh, Pennsylvania, CHASM is a multidisciplinary effort of doctors, residents, and students aimed at providing basic medical care with dignity and developing meaningful relationships.^21^ CHASM provides biweekly outreach services including hygiene supplies, clothing, and basic healthcare screenings to individuals living in shelters and on the streets.

This research seeks to explore how members of Charleston’s homeless community perceive their health and interactions with the healthcare system, with the goal of informing more equitable, compassionate, and effective interventions. By seeking direct input from the homeless population in this small Appalachian city, we aimed to better understand potential biases and perceived discrimination that may contribute to hesitancy to seek appropriate medical care and influence healthcare utilization in a group of people facing barriers to health care that are both systemic and multifaceted.

## METHODS

### Participants

This survey study was approved as exempt under 45 CFR 46/21 CFR 56 by the West Virginia University Charleston Division Institutional Review Board. Participants were recruited from select temporary housing shelters and residential facilities in Charleston, WV. Recruitment was conducted by third- and fourth-year medical students who are members of CHASM. Participants were approached during pre-established bi-monthly outreach visits to three local shelters: the YWCA Sojourner’s Shelter for Homeless Women and Families (which houses single women, families, and men with children), the Union Mission Men’s Shelter (which houses adult men), and the Roark Sullivan Lifeway Center (which also serves adult men). Inclusion criteria included: (1) being 18 years of age or older; (2) current residence in one of the aforementioned shelters visited by CHASM-affiliated medical students; (3) the ability to understand and speak English; and (4) willingness to participate in the survey.

### Data Collection

A cross-sectional study methodology was chosen due to ease of execution and accessibility of population of interest through the existing CHASM program. Additionally, a cross-sectional study would not rely on patient follow-up, which would have been difficult in this population. The cross-sectional study design carries low risk to this vulnerable population while providing data that can be used to inform further program or research efforts.

Data were collected via a voluntary, anonymous survey administered using REDCap on password-protected iPads. CHASM student members distributed the survey in person at the three participating shelters and in adjacent street areas. Study personnel provided a verbal explanation of the study, emphasizing the voluntary and anonymous nature of participation. A waiver of documentation of consent was obtained to preserve anonymity. The survey began with a cover letter that included an IRB-approved altered consent; signatures were not requested. Data collection began in September 2023 and continued through March 2024.

Survey items (Table 1) were developed by study investigators who participated in the CHASM project and were guided by their experience serving patients and their interest in the population. The survey first queried demographic data including age, gender, race, and current housing and employment status, with optional questions on substance use disorders, mental health diagnoses, and history of incarceration. Subsequent questions explored healthcare experiences and perceptions of stigma related to housing status. These were assessed using a 4-point Likert scale.

### Confidentiality

Subject confidentiality was strictly maintained via an anonymous survey. Patient data was obtained through a secure login system. All patient information was stored on a password-protected computer and a password-protected file. The data is only accessible to the study investigators.

### Data Analysis

Descriptive analysis was conducted for each variable in this study. Means and standard deviations were reported for continuous variables, and frequencies and proportions were calculated for categorical variables. To assess statistically significant associations, chi-square or Fisher’s exact tests were applied to the categorical variables, depending on cell sizes. A significance level of *p* < 0.05 was used for all comparisons. Analyses focused on associations between healthcare experiences and utilization, as well as relationships between demographic characteristics and health-related attitudes. All analyses were conducted using SAS Studio 3.8.

## RESULTS

A total of 108 participants completed the survey (Table 2). The mean participant age was 45.8±12.4 years. Most participants identified as white (n=70, 64.8%); 20.4% identified as black or African American (n=22) (Table 2) The majority identified as cisgender men (n=71, 65.7%) or cisgender women (n=34, 31.5%). Most participants reported being homeless or living in a shelter (76.6%). Only 21.3% reported full-time employment. Over half of the participants (n=62, 57.4%) were not working stating that they were either not able to work (n=27, 25%), unemployed and looking for work (n=25, 23.2%) or not looking for work (n=3, 2.8%), or retired (n=7, 6.5%). Half (n=55, 50.9%) reported a mental health diagnosis (including depression, bipolar disorder or schizophrenia), and 44.3% reported a history of substance use.

Of 108 survey respondents, 25 (23.2%) “agreed” or “strongly agreed” that they feel judged by healthcare workers at the hospital, and 25 (23.2%) “agreed” or “strongly agreed” that they feel healthcare workers treat them differently from the non-homeless people in the hospital (Table 3). Similarly, 21 (19.4%) agreed/strongly agreed that they do not like going to the hospital because of the way they are treated, and 17.6% reported distrust in healthcare workers due to past negative experiences. While 86.9% of respondents (n=93) agreed that their health is important, only 71.3% felt their health problems were well controlled (n=77). Although a majority (n=95, 88.0%) felt respected by healthcare workers, 25 (23.2%) reported feeling judged or treated differently due to their housing status.

Participants who reported a mental health diagnosis of depression, bipolar disorder, or schizophrenia reported significantly higher levels of stigma or poor experience with providers (Table 4). A significant proportion of participants who reported a mental health diagnosis disagreed with the question “Doctors, nurses, and healthcare workers treat me with respect at the hospital.” (*p* =0.026) and agreed with the statement “I feel like doctors, nurses, and healthcare workers treat me differently from the non-homeless people in the hospital” (*p* =0.027) (Table 4). Additionally, there was some indication that homeless individuals who identified as a gender minority had a greater perceived stigma (Table 4).

## DISCUSSION

When assessed using a survey administered by medical students who regularly interacted with the homeless community, perceived stigma and inequity within healthcare settings were prevalent among people experiencing homelessness in Charleston WV, a small Appalachian city. This study adds to a limited body of U.S.-based research and even more limited body of Appalachian-based research exploring how individuals experiencing homelessness perceive stigma and inequity when interacting with the healthcare system. Consistent with prior studies from other countries and other regions of the U.S., a substantial proportion of the survey respondents reported feelings of judgment and stigmatized treatment by healthcare professionals. Importantly, these perceptions were not evenly distributed across all individuals surveyed, and homeless individuals who self-reported a history of depression, bipolar disorder, or schizophrenia were significantly more likely to report stigma or dissatisfaction with their healthcare experience.

Homelessness is known to comprise many different living conditions. These include sheltered and unsheltered homelessness, which are also referred to houselessness and rooflessness, as well as living in insecure or inadequate conditions.^22^,^23^ Individuals in these situations also may have unmet basic psychological needs.^24^ While this study allowed participation of individuals experiencing any type of homelessness, the recruitment of participants at homeless shelters and from nearby areas likely skewed the cohort toward sheltered homeless individuals, and the questionnaire did not differentiate between sheltered and unsheltered individuals.

Residents of Appalachian states, including WV, face significantly worse healthcare outcomes than the residents of other regions due to a complex interplay of infrastructural and systemic disparities.^25^–^27^ These include a shortage of healthcare providers, a lack of accessible mental health resources, and geographic isolation that increases the distance between patients and medical services.^18^,^26^ In WV specifically, widespread mistrust of the healthcare system persists, rooted in a history of overprescription that contributed to the opioid epidemic and compounded by generally low levels of healthcare literacy.^28^ These factors create substantial and persistent barriers to care across the region, which extend to the homeless population.

Poor physical health and mental health are prevalent among the homeless population in West Virginia. A study conducted by the WV Department of Human Services Bureau for Behavioral Health using data from the state Homelessness Management Information System (HMIS) found that among WV residents experiencing sheltered or unsheltered homelessness, 18% (~ 1 in 5) reported having a physical disability and 14% reported having a chronic health condition. 29 Among HMIS clients from 2018–2023, 35% reported a mental health disorder, which is higher than estimates for the general population of WV and suggested a high burden of mental health disorders in the WV homeless population.^29^ The perceptions reported by homeless individuals with a mental health diagnosis in our cohort further expose a particularly vulnerable population. The finding of a history of depression, bipolar disorder, or schizophrenia in 50% of our cohort and the association of these self-reported mental health disorders with bias and stigmatization in healthcare settings underscores the potential importance of improving services focused on these individuals and targeting educational efforts to their providers.

The proportion of men among the survey respondents was slightly higher than that observed for WV as a whole or for the 4-county Kanawha Valley Collective Continuum of Care (KVC CoC), which contains the City of Charleston but is considered largely suburban, in the 2024 Point-in-Time study.^16^,^30^ Our distribution of 66% men, 32% women, and 1.8% gender-diverse was similar but not identical to the Point-in Time state distribution of 61% men, 38% women, and 1.5% gender-diverse and the KVC CoC distribution of 62% men and 37% women.^16^,^30^ However, the Point-in-Time study found that only 0.5% of homeless individuals in the KVC CoC were gender-diverse, as compared with 1.8% in our cohort.^30^ This suggests that further study of relationships between homelessness and healthcare in gender-diverse individuals in WV might be most effective if conducted in a urban setting rather than in the CoC regions or the state as a whole. State-specific study of impacts of homelessness on healthcare in gender-diverse individuals is likely necessary given state-to-state variability in legislation affecting this population.

The proportion of non-white individuals among the survey respondents was also slightly higher in the Point-in-Time study for WV as a whole or for the KVC CoC. Individuals who identified as American Indian/Alaskan Native or Native Hawaiian/Pacific Islander comprised 2.8% and 4.6% of our respondents, respectively, but were <1% of homeless individuals in WV identified in the Point-in-Time study.^16^,^30^ The proportion of black/African American in our survey was also higher than anticipated by the 2024 Point-in-time study for West Virginia or the KVC CoC (20% vs 11% and 16%, respectively) and was also high relative to the racial demographics of the City of Charleston (12% black/African American).^16^,^30^,^31^ As a result, the proportion of white individuals surveyed was relatively lower (65% of our cohort vs. 83% of the Point-in-Time WV cohort, 78% of KVC CoC Point-in-Time cohort, and 76% of the Charleston population). 30, 31 One trend observed in our cohort was that non-white homeless individuals mistrusted healthcare workers more frequently than white homeless individuals, which suggests that the City of Charleston may be an appropriate locale to study the intersection of race, healthcare interactions, and homelessness in WV, and perhaps in Appalachia. While not statistically significant in this pilot study, perceptions reported by homeless individuals identifying as racial minorities may reflect clinically relevant differences in the population that could be addressed.

Limitations to the study include a limited geographic distribution of survey participants to individuals residing in or near shelters in Charleston WV, which may not be generalizable to homeless populations in other Appalachian locales or those who avoid shelters altogether. We did not examine the citizenship status of the individuals surveyed, therefore potential confounding by this factor could not be examined. The use of self-reported survey data also introduced the possibility of recall or response bias. Additionally, due to the small sample size, our ability to stratify the results by subgroup for multivariate analysis was limited. Further research is needed with larger sample sizes to allow for multivariate analyses of the factors contributing to dissatisfaction with care among homeless individuals in WV. Additionally, longitudinal studies examining how perceptions of stigma impact healthcare utilization and long-term outcomes in homeless WV residents are essential for designing sustainable, region-specific interventions.

## IMPLICATIONS

Our findings point to a troubling reality: some of the most medically vulnerable individuals may delay or avoid necessary care due, in part, to prior negative interactions with healthcare providers. This avoidance contributes to increased rates of preventable morbidity and mortality within the homeless population. These results resonate with both regional and national concerns about healthcare disparities among marginalized and socioeconomically disadvantaged groups. In Appalachian states—where chronic disease is a leading contributor to premature death—the intersection of housing insecurity with other social determinants of health creates compounding barriers to effective care.^18^,^26^ Efforts to improve healthcare access and quality for homeless individuals must include interventions to reduce provider bias, foster trust between clinicians and patients, and incorporate the voices of sheltered and unsheltered homeless individuals, as well as individuals with insecure or inadequate housing, into care design. These interventions must consider region-specific factors. Healthcare models that prioritize patient-centered care and continuity with primary care providers are potential strategies to improve the patient experience and health outcomes. 32, 33

**SUMMARY BOX**

**What is already known about this topic?**

People experiencing homelessness often face stigma in healthcare settings, which can lead to avoidance of care and poorer health outcomes. Most existing research on this issue comes from outside the U.S.

**What is added by this report?**

This study highlights that perception of stigma and inequitable treatment when interacting with the health care system are prevalent among the homeless population in Charleston WV, particularly among those with mental health conditions.

**What are the implications for future research?**

Larger, more diverse studies are needed to explore how stigma affects healthcare use and outcomes. Future research should also evaluate interventions that reduce provider bias and build trust with individuals experiencing homelessness.

## Figures and Tables

**Table 1:** Questions Included in Survey.

A. Demographic Questions
1). How old are you?

2). What is your gender?
[Man (cisgender); Woman (cisgender); Transgender woman; Transgender man; Gender nonbinary; Gender diverse; Agender/No gender; I prefer a different term to describe my gender identity]

3). Which of these groups would you say best represents your race?
[White; Black or African American; Asian; American Indian or Alaska Native; Native Hawaiian or Pacific Islander; Other; Prefer not to answer]

4). What best describes your current living situation?
[Live alone in my own home (house, apartment, condo, trailer, etc.), may have a pet; Live in a household with other people; Live in a residential facility where meals and household help are routinely provided by paid staff (or could be if requested); Temporarily staying with a relative or friend; Temporarily staying in a shelter or homeless; Other]

5). What is your current employment status?
[Employed full-time; Employed part-time; Unemployed- Looking for work; Unemployed- Not looking for work; Homemaker; Military/Forces; Retired; Not able to work; Other]

**B. Optional Demographic Questions**

1). Check any of the following that apply:
- I currently have problems caused by drinking alcohol or using drugs that weren’t prescribed to me.
- In the past, but not now, I had problems caused by alcohol or using drugs that weren’t prescribed to me.
- I have never had problems caused by alcohol or using drugs that weren’t prescribed to me.
- Prefer not to answer

2). Have you been incarcerated in the last year?
- Yes
- No
- Prefer not to answer

3). Have you been diagnosed with depression, bipolar disorder, or schizophrenia?
- Yes
- No
- Prefer not to answer

**C. Healthcare attitudes and perceived stigma questions**
*(4-point Likert Scale, Strongly Agree to Strongly Disagree)*

1). My health is important to me.

2). I feel like my health problems are well controlled.

3). Doctors, nurses, and healthcare workers treat me with respect at the hospital.
4). I feel judged by doctors, nurses, and healthcare workers at the hospital.

5). I feel like doctors, nurses, and healthcare workers treat me differently from non-homeless people in the hospital.

6). I do not like going to the hospital because of the way I am treated there.

7). I do not trust healthcare workers because of bad past experiences.

**Table 2:** Demographic Information of Survey Respondents

Characteristic^*^	Frequency, n (%)
Age, mean (min, max,)	45.8 (21.0, 75.0)
**Race**	
White	70 (64.8%)
Black or African American	22 (20.4%)
American Indian or Alaskan Native	5 (4.6%)
Native Hawaiian or Pacific Islander	3 (2.8%)
Other	6 (5.6%)
Prefer not to answer	2 (1.8%)
**Gender**	
Man (Cisgender)	71 (65.7%)
Woman (Cisgender)	34 (31.5%)
Man (Transgender)	0 (0%)
Woman (Transgender)	1 (0.9%)
Gender nonbinary	1 (0.9%)
I prefer a different term	1 (0.9%)
**Housing Status**	
Temporarily staying in a shelter or homeless	82 (76.6%)
Live in a residential facility where meals and household help are routinely provided by paid staff (or could be if requested)	15 (14.0%)
Live alone in my own home (house, apartment, condo, trailer etc.); may have a pet	5 (4.7%)
Live in a household with other people	2 (1.9%)
Other	3 (2.8%)
**Employment**	
Employed full-time	23 (21.3%)
Employed part-time	9 (8.3%)
Unemployed-Looking for work	25 (23.2%)
Unemployed- Not looking for work	3 (2.8%)
Not able to work	27 (25.0%)
Retired	7 (6.5%)
Homemaker	1 (0.9%)
Other	13 (12.0%)
**Incarceration Status**	
No	82 (75.9%)
Yes	22 (20.4%)
Prefer not to answer	4 (3.7%)
**Mental Health Diagnosis**	
*Have you been diagnosed with depression, bipolar disorder, or schizophrenia?*	
Yes	55 (50.9%)
No	49 (45.4%)
Prefer not to answer	4 (3.7%)
**Substance Abuse History**	
I have never had problems caused by drinking alcohol or using drugs that weren't prescribed to me.	47 (44.3%)
In the past, but not now, I had problems caused by drinking alcohol or using drugs that weren't prescribed to me.	35 (33.0%)
I currently have problems caused by drinking alcohol or using drugs that weren't prescribed to me.	8 (7.6%)
Prefer not to answer	16 (15.1%)

NOTES:

a Data are given as n (%) unless indicated otherwise.

**Table 3:** Survey Responses to Perceived Stigma in Healthcare Settings

Statement	Strongly Agree (n)	Agree (n)	Disagree (n)	Strongly Disagree (n)	Total Agreement (n) (%)	Total Disagreement (n) (%)
My health is important to me.	58	35	2	12	93 (86.9%)	14 (13.1%)
I feel like my health problems are well controlled.	22	55	19	12	77 (71.3%)	31 (28.7%)
Doctors, nurses, and healthcare workers treat me with respect at hospital.	50	45	7	6	95 (88.0%)	13 (12.0%)
I feel judged by doctors, nurses, and healthcare workers at the hospital.	11	14	40	43	25 (23.2%)	83 (76.9%)
I feel like I’m treated differently from non-homeless people.	15	10	48	33	25 (23.2%)	81 (75.0%)
I do not like going to the hospital because of the way I am treated there.	9	12	47	39	21 (19.4%)	86 (79.6%)
I do not trust healthcare workers because of bad past experiences.	6	13	47	42	19 (17.6%)	89 (82.4%)

**Table 4:** Perceived Stigma amongst Respondents Stratified by Demographic Subgroup

Characteristic a	Q8. Doctors, nurses, and healthcare workers treat me with respect at the hospital	Q9. I feel judged by doctors, nurses, and healthcare workers at the hospital	Q10. I feel like doctors, nurses, and healthcare workers treat me differently from the non-homeless people in the hospital	Q11. I do not like going to the hospital because of the way I am treated there	Q12. I do not trust health care workers because of bad past experiences
Gender	*p* =0.17	*p* =0.37	*p* =0.058	*p* =0.087	*p* =0.27
Race	*p* =0.36	*p* =0.34	*p* =0.44	*p* =0.27	*p* =0.064
Incarceration	*p* =0.58	*p* =0.23	*p* =0.36	*p* =1.00	*p* =1.00
History of Mental Health Diagnosis	*p* =0.026	*p* =0.55	*p* =0.006	*p* =0.19	*p* =0.35
Employment Status	*p* =0.74	*p* =0.25	*p* =0.59	*p* =0.73	*p* =0.62
Housing Status	*p* =0.15	*p* =0.75	*p* =0.31	*p* =0.29	*p* =0.16

NOTES:

a Fishers exact test was used to determine if question responses differed significantly by demographic characteristic.

b Gender was dichotomized for analysis to include cisgender male and cisgender female vs. all other gender identities.
